# Perceptions of and Responses to Wildfire Smoke Among New York State Residents: A Cross-Sectional Study

**DOI:** 10.3390/ijerph22020277

**Published:** 2025-02-14

**Authors:** Erandy I. Barrera, Alistair Hayden, Genevive Meredith, Corinna A. Noel

**Affiliations:** Department of Public and Ecosystem Health, Cornell University, Ithaca, NY 14850, USA; eib34@cornell.edu (E.I.B.); ath79@cornell.edu (A.H.); gmeredith@cornell.edu (G.M.)

**Keywords:** wildfire smoke, air quality, health, preventative action

## Abstract

Exposure to wildfire smoke (WFS) is associated with detrimental physical and mental health. Periods of sustained WFS are predicted to increase with climate change, affecting populations globally. Using a retrospective cross-sectional study, we assessed perceptions of and responses to WFS in a cohort of New York State (NYS) residents in Summer 2023. Data were collected using an online survey from October to November 2023. Descriptive statistics summarized respondent experiences, while exploratory analyses identified high-risk populations using chi-square and *t*-tests. Our sample consisted of 609 primarily healthy, white, and well-educated individuals who spent most of their time in NYS during Summer 2023. Of the 99% that reported experiencing WFS, 92% received and 91% sought out WFS-related air quality information. While only 25% reported a WFS-related illness, 87% experienced at least one symptom with WFS, frequently citing watery eyes (63%), irritated throat (50%), and headaches (49%), with women reporting symptoms more frequently than men (89.1% vs. 81.6%; *p* = 0.034). A majority (93%) reported taking mitigation actions, including avoiding outdoor activities (75%) and wearing masks (54%). Our results highlight widespread impacts of wildfires in NYS during Summer 2023, with nearly all respondents reporting sustained periods of WFS. Most reported at least one adverse health symptom despite taking preventative measures, indicating that current protective strategies may be insufficient and more effective interventions are needed.

## 1. Introduction

The adverse health effects associated with exposure to wildfire smoke (WFS) represent a growing public health issue in both the United States (US) and globally. A warming climate, increases in frequency of drought and extreme weather events, human activity within the wildland–urban interface, and fire suppression practices over the last century have been suggested to contribute to the severity and frequency of wildfires, resulting in more populations exposed to WFS than ever before [1,2,3,4]. In the coming decades, prolonged periods of WFS are predicted to increase, including in the northeastern region of the US [1,2].

WFS is hazardous to health as particulate matter and bioaerosols are introduced into the air, worsening air quality [5]. Particulate matter smaller than 2.5 μm in diameter (PM_2.5_) are of particular concern because the small particles enter the respiratory system and deposit deep in lungs [6,7]. While PM_2.5_ is emitted via multiple sources, such as vehicles, home heating, and recreational camp fires, the PM_2.5_ in WFS may be more harmful to health due to its biochemical composition [8,9]. Health impacts from WFS include headaches, irritated sinuses, asthma, and chest pain, with short-term exposure associated with increased hospitalizations for respiratory and psychological conditions like asthma and anxiety [5,6,7,10,11,12,13]. Additionally, PM_2.5_ can increase mortality risk by exacerbating pre-existing health conditions among certain sensitive groups, including older adults, young children, pregnant women, and individuals with heart and lung conditions [11,14,15].

To date, most US-based wildfire and WFS research has focused on western states, a region with a historically higher wildfire burden; however, with climate change, wildfires are affecting more communities across North America [16,17,18,19,20,21]. For a six month period from April to September of 2023, wildfires burned in each Canadian province spanning from British Columbia to Quebec [22]. Approximately 15.0 Million hectares (Mha) of land was burned, seven times higher than the annual average rate (2.1 Mha) [22]. As a result, record high levels of carbon emissions were documented. 763 Tg of carbon (Tg C) was released, which was five times higher than the previously recorded high of 152 Tg C in 2021 [23]. Resulting WFS plumes traveled down the northeastern seaboard and blanketed the northern and southern US [22]. States such as New York, Pennsylvania, and Georgia experienced sustained periods of elevated PM_2.5_ levels above the “healthy” level set by the U.S. Environmental Protection Agency (EPA) [22]. In New York, public alerts were issued by the New York State Department of Environmental Conservation and Department of Health on June 2, 2023 for ozone and on June 5 and 6 for fine particulate matter, but it is unclear how many people were aware of these alerts [24]. Some jurisdictions took actions as well; for example, the mayor of New York City issued statements starting the evening of June 6 and held press conferences starting June 7 [24]. Given the intensity of wildfires in Summer 2023, as well as the novel level of sustained exposure to WFS in the northeastern US, we sought to explore how a cohort of New York State (NYS) residents perceived WFS, accessed air quality information, experienced its effects on physical and mental health, and adopted protective measures during this period [25,26,27].

## 2. Materials and Methods

A descriptive cross-sectional survey, administered via Qualtrics (Version 2005, 2024 Qualtrics, Provo, UT, USA) was used to gather data on the following research questions related to respondents’ experiences with sustained periods of WSF during Summer 2023:

(1) How was information on air quality accessed and received?
(2) Did individuals perceive wildfire smoke as a health hazard?
(3) What were the self-reported health impact(s) of wildfire smoke?
(4) What behavioral measures were taken to mitigate negative health impacts?

The study was approved as exempt by Cornell University’s Institutional Review Board (IRB0147809), and all participants provided informed consent.

### 2.1. Survey Design

To maximize reliability, validity, and ease of comparison, our survey was adapted from Fowler and colleagues’ 2019 study of perceptions and responses to WFS among northwestern US residents, which focused specifically on the Boise, Idaho Metropolitan Area [28]. We used 23 of 29 survey questions that were in line with our research questions and added 7 new questions examining outdoor activity during the smoke wave and sources of air quality information and notifications. We collected information about age, gender, race/ethnicity, income level, and geographical location at the time of the Summer 2023 wildfires, as well as perception of WFS as a natural hazard, overall health status, symptoms experienced, and protective actions taken. Our survey employed various categorical question types, including multiple-choice, Likert scale, and dropdown options. Some questions included a “not sure” option, an “other” option with a text box for additional responses, or a text box for elaboration alongside the “yes” option (Supplemental File S1).

To maintain data integrity, we activated the following security measures in Qualtrics: within question verification of geographic location, bot detection, security scan monitors, relevant ID, and indexing prevention. The survey prompted respondents to reflect on their experiences during the 2023 smoke wave, was pilot tested before use, and took approximately 10 min to complete. When participants finished the survey, they could choose to enter a raffle for one of five USD 50 Amazon gift cards.

### 2.2. Data Collection

Using a convenience sampling approach, participants were recruited via emails from trusted leaders (NYS public health agencies, Cornell University, and Cornell Cooperative Extension) and via social media. Interested respondents over the age of 18 years completed the online survey in English using Qualtrics. Data were collected from October 2023 to November 2023.

Through recruitment, a total of 1246 survey responses were collected. However, responses were excluded from final analysis if they met any of the following exclusion criteria:

(1) Respondent listed a residence outside of NYS during Summer 2023;
(2) Qualtrics system flagged a response as a bot;
(3) Qualtrics system flagged a response as a duplicate entry;
(4) Fewer than 50% of the survey questions were answered;
(5) Respondent spent less than 120 s on the survey;
(6) The state, county, or city provided did not match any location within NYS; or
(7) State and county or zip code with city was not provided.

After applying all exclusion criteria and cross-checking geographical locations using zip codes and municipality names, our final sample consisted of 609 responses. Responses were recorded exactly as they were received. To visualize the geographical distribution of responses within NYS, a density dot map was created using ArcGIS^®^ Pro 3.30 (2024 Esri Inc., Redlands, CA, USA).

### 2.3. Data Analysis

Descriptive statistics including response count and percentage were calculated using Stata 18 (2023 StataCorp LLC, Stata Statistical Software: Release 18. College Station, TX, USA). Answers from open response “other” options were recoded to fit into existing response options, or a new category was created if common words or phrases were identified. As participants were able to skip any question they did not want to answer, some questions were missing responses. We report percentages that were calculated after excluding missing or unknown responses. Respondents included in our study provided responses to 97% to 100% of the survey questions.

Using chi-square, Fisher’s exact tests, and *t*-tests, exploratory analyses aimed to identify high-risk populations by examining the association between respondent characteristics (age, income, race/ethnicity, gender, and self-reported health status) and reported prevalence of:

(1) At least one wildfire smoke-related symptom;
(2) The most common symptoms (itchy/irritated/watery eyes, sore/irritated throat, and headaches)
(3) Taking any preventative action during smoke events; and
(4) Taking future preventative action.

A table summarizing the results of these exploratory analyses is provided in the main text. For further detail, please see Supplemental Tables S1–S6. A threshold of *p* < 0.05 was used to determine statistical significance.

## 3. Results

### 3.1. Study Population

**Figure 1:**
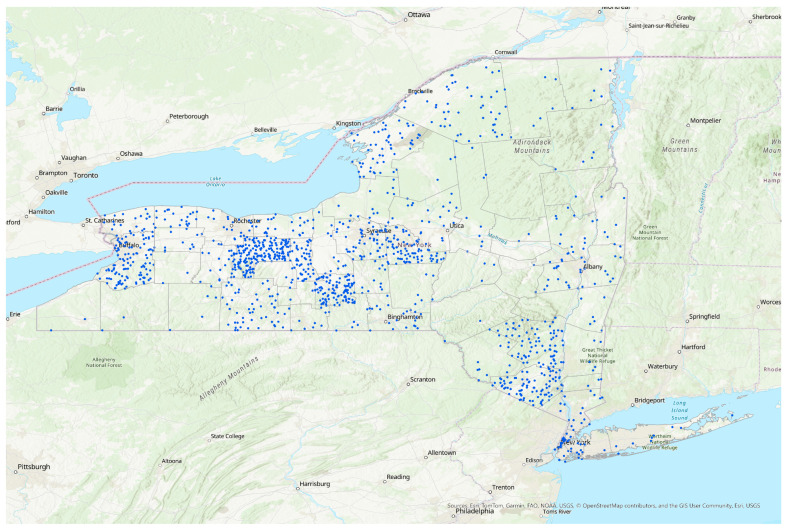
Distribution of respondents across New York State. Each dot represents one respondent in our study.

Survey responses (*n* = 609) were collected from individuals across the state, with clusters in central New York, select metropolitan areas (e.g., New York City and Buffalo) and primarily rural regions (Figure 1). Nearly all (99%) individuals reported experiencing one or more days of poor air quality due to WFS during Summer 2023, with 25% experiencing seven or more consecutive days of poor air quality in their area (Table 1). Given our convenience sampling approach, our study population was not representative of the population of New York State, consisting of primarily well-educated, white women ranging between 18 and 44 years, with overall excellent or good health status [29]. The majority (59%) of respondents reported an annual income between USD 50,000 and USD 150,000, but incomes ranged from less than USD 25,000 to USD 250,000 or more. Approximately 90% of our study population reported engaging in outdoor leisure activities during Summer 2023, but only 37% of these participants engaged in outdoor activity daily. 46% of all respondents stated being required to work outdoors fully exposed to WFS and other weather conditions, but only 9% of outdoor workers reported needing to do so every day.

### 3.2. Sources of Air Quality Information During Summer 2023

A total of 92% of respondents reported receiving air quality notifications during Summer 2023 (Table 2). Common sources of information included smartphone apps (78%) and local health agencies like the county health department (53%). Additionally, 91% sought out their own information regarding wildfires and smoke events, with 65% utilizing smartphone apps, 59% personal observation, and 59% online news sources. During any smoky week in the summer, 56% reported looking online via a computer, tablet, or smartphone to seek out smoke-related information, smoke forecasts, and health notices for 6 days or more.

### 3.3. Perceptions of Wildfire Smoke as a Natural Hazard

A total of 85% of respondents considered WFS events a natural hazard (Table 3). However, when asked about evacuating a home due to WFS, 60% said they would, 19% said they would not, and 19% were unsure of the decision to evacuate.

### 3.4. Self-Reported Impacts of Wildfire Smoke on Health

In our sample, 25% reported experiencing a WFS-related illness during Summer 2023 but 87% reported one or more adverse health symptoms during periods of WFS (Table 4). Of people reporting symptoms, 61% reported no WFS-related illness and 13% reported being unsure if they experienced a WFS-related illness. Commonly reported physical health symptoms included itchy/irritated/watery eyes (63%), a sore or irritated throat (50%), and headaches (49%), as well as mental health impacts such as fatigue (21%) and anxiety (20%). Some respondents reported other symptoms such as rashes, concerns about pet health, and feeling “off” with no explanation.

Exploratory analyses, as summarized in Table 5, suggest that women tended to report symptoms more frequently than men, particularly for any symptom (89.1% vs. 81.6%; *p* = 0.034), headaches (58.4% vs. 32.1%; *p* < 0.001), and sore or irritated throat (58.2% vs. 45.2%; *p* = 0.028). While symptom prevalence didn’t vary consistently by self-reported health status, individuals with excellent health did report fewer headaches during times of WFS compared to those with poorer health (43.6% vs. 59.1%; *p* = 0.001). Additionally, respondents with yearly incomes below USD 25,000 reported more itchy, irritated, or watery eyes compared to those with higher incomes (93.1% vs. 70.8%; *p* = 0.017) and those identifying as non-Caucasian cited fewer sore or irritated throats than Caucasians (37.2% vs. 57.0%; *p* = 0.013), although the sample size was limited (*n* = 16) among racial/ethnic minorities reporting this symptom.

### 3.5. Mitigation Actions to Protect Health

Most respondents (93%) reported taking one or more mitigation actions during WFS periods (Table 6). Of the respondents that took at least one mitigation action, the most frequently utilized actions were avoiding outdoor leisure activities (75%), wearing a mask (54%), and using a personal air filtration system (34%). Other mitigation actions noted through open text responses included intentionally changing breathing techniques, rescheduling surgeries or other medical operations, and using herbal remedies to reduce irritation. Exploratory analyses suggest that mitigation actions were largely consistent across age, race/ethnicity, gender, health status, and income levels (*p* > 0.05; see Table 5).

When asked about the longest period of consecutive days in which outdoor activity was reduced or eliminated, 41% reported a 2- or 3-day period, and 16% reported reducing activities for 6 days or more. Additionally, when asked what information motivated the decision to reduce or eliminate activity, 80% reported using both personal observations and air quality information from local, state, or federal sources. Some respondents reported reducing or eliminating activity due to advice from social media or past experiences with breathing troubles.

Most respondents (50%) reported that they would reduce outdoor activity on a given day when the Air Quality Index (AQI) reached the orange level, indicating air quality that is “unhealthy for sensitive groups”, such as immunocompromised individuals. 41% of respondents stated they would eliminate outdoor activities entirely at the red AQI level (“unhealthy” air quality), though 34% reported eliminating all their outdoor activities at the orange level. Overall, for most of our study population, activity reduction was triggered at AQI levels from yellow to red (“moderate” to “unhealthy”), while activity elimination occurred at higher AQI levels, ranging from orange to purple (“unhealthy for sensitive groups” to “very unhealthy”).

Finally, respondents indicated their willingness to take preventative action to reduce future smoke-related health impacts, with 62% stating they would and 32% expressing uncertainty. Intention to take future preventative action did not substantially differ according to age, gender, race/ethnicity, income, or health status (Table 5). In open-response options, some respondents reported that they would stay indoors, wear a mask when going outside, use air filtration systems, and stay up to date with weather alerts for future smoke events. Other respondents reported that they would eat healthy and maintain a strong immune system. One respondent said they would contribute to the fight against climate crises and climate change, while another said they would not live in wildfire-prone areas.

## 4. Discussion

In this study, we aimed to explore perceptions, health impacts, and responses to prolonged periods of WFS exposure among NYS residents during Summer 2023. Almost all individuals surveyed reported experiencing poor air quality due to WFS and receiving or seeking out smoke-related information during this time. Despite perceiving smoke as a hazard and taking mitigation actions to reduce their own exposure, most reported at least one adverse health symptom. Our study fills a gap in the literature by providing perspectives of a cohort of an eastern US population, who, until recently, had not experienced prolonged periods of WFS exposure.

### 4.1. Reliance on Smartphone Applications as a Source of Air Quality Information

We show that approximately 9 in 10 respondents living in NYS during Summer 2023 obtained air quality information in times of WFS, with most utilizing smartphone applications to stay informed. Compared to a similar study conducted in northwestern US in 2018, more participants in our study sought out (91% vs. 65%) and received (92% vs. 67%) WFS-related notifications, suggesting that this type of environmental information is potentially more accessible than it was 5 years ago, coinciding with increased smoke awareness and technological advances [28]. In the same study, the use of smartphone applications was not commonly cited by mostly college-aged respondents, who instead relied on a variety of online sources (19%), personal observation (16%), television (11%), friends and family (10%), and social media (10%) [28]. It is possible that these discrepancies could be attributed to motivational differences in information seeking behavior related to WFS, demographic variations between our study populations, or the recent development of smartphone applications for air quality.

In the past, television and radio have been largely used as sources for alerts related to environmental conditions [30]. However, our findings and other literature show increased usage of smartphone applications and social media as sources of environmental information by younger individuals [30]. Smartphones applications have advantages over television and radio, as information is rapidly available to consumers at any time of the day in a compact format. Additionally, some smartphone applications allow users to report their own WFS experiences in real time, offering added value to the information consumers are able to access [31,32]. For example, the EPA created ‘Smoke Sense’, an application that lets citizen scientists use their smartphones to view and track air quality, learn mitigation actions, and provide their own experiences about the smoke [32]. While smartphone applications can aid in sharing information more rapidly and efficiently, they are often only accessible to individuals who have a smartphone, have downloaded a relevant app, know how to use it, and understand the messaging provided within the app [33].

### 4.2. Reported Symptoms During Smoke Events

Our findings reveal minor, but widespread reports of adverse symptoms experienced during WFS events, at least in our sample of NYS residents during the 2023 Canadian wildfires. Despite representing a generally healthy population, 87% reported at least one adverse symptom during periods of smoke, with headaches, sore/irritated throat, and itchy/watery eyes as the most frequently cited. Women reported a higher prevalence of adverse health symptoms, in line with previous work suggesting a sex-specific difference in health with exposure to WFS [34]. Symptom prevalence did not consistently vary with self-reported health status, suggesting that poor air quality from WFS, rather than pre-existing conditions, contributed to the reported symptoms. The common symptoms that we observed were also reported in a recent study examining health impacts of WFS in Idaho, but at a lower prevalence [28]. As wildfire-specific PM_2.5_ affects the respiratory and cardiovascular system, it is reasonable that prolonged exposure may result in headaches, irritated sinuses, chest pain, and asthma [7]. The self-reported increase in health impacts reported here mirrors the increase in health-service usage recorded in New York State during the smoke waves, including increases in 9-1-1 calls for respiratory complaints in New York City [35] and asthma emergency-department visits statewide [13,36].

We also find that mental health may be adversely affected during times of smoke, with nearly one in four respondents in our study reporting anxiety, stress, or fatigue. While the impacts of WFS on physical health has been well studied, less is known about mental health [37,38]. Recent work suggests that exposure to WFS is associated with increased emergency department visits for anxiety disorders and psychotropic prescriptions such as antidepressants and mood stabilizers, with strongest effects observed in women and older adults [12,39]. Some research attributes adverse mental health effects of air pollution to the isolation and reduction of physical activity that comes from limiting outdoor activities during times of poor air quality, which we also observed in our study [40]. Future work should further explore the mental health effects of WFS as well as availability of services and public health interventions designed to mitigate effects.

Interestingly, we found that only 25% of respondents reported a smoke-related illness, yet nearly 90% reported at least one symptom during periods of WFS exposure. Of those reporting symptoms, 61% stated they did not experience a WFS-related illness. This is in line with a finding from Fowler and colleagues who surveyed primarily Boise, Idaho residents after a prolonged period of WFS in 2019 [28]. Illness perception can differ among individuals, influencing if an individual reports a symptom rather than a defined illness. Looking at the psychological aspects of illness perception, Leventhal et al. (1984) identified a five-part model regarding how symptom presentation may influence cognitive and emotional response to illness [41,42]. Model components included identity of symptoms related to the illness, consequences, curability, expected illness duration, and emotional response [41,42]. An individual might not perceive a WFS-related illness if at least one of these components is not explicitly defined for an illness caused by smoke. For example, an illness caused by a pathogen such as viral influenza has defined symptoms, consequences, curability, and a known illness duration. Individuals understand the various components that identify viral influenza as an illness. This may not be the case for WFS-related illness. Additionally, individuals with pre-existing conditions may attribute new symptoms to an exacerbation of their condition, rather than a new smoke exposure-related illness, although this was likely not the case in our sample as only 11.5% of respondents reported fair or poor general health.

### 4.3. Mitigation Actions During Smoke Events

In our survey, 93% of respondents reported using at least one mitigation action to protect their health during a smoke wave in Summer 2023, including reducing or avoiding outdoor activities, wearing a mask, and using an air filtration system. Most of the actions taken align with current guidelines provided by the Center of Disease Control (CDC) and the NYS Department of Health (DOH) [43]. A similar proportion of medically vulnerable adults in California took action to protect themselves from WFS during periods of prescribed burns [44]. Fowler et al. (2019) reported that participants commonly took medications to mitigate health impacts during times of WFS, but fewer used an air filtration system as compared to our sample [28]. This discrepancy could potentially be explained by study timing, as we conducted our study following the COVID-19 public health emergency. During the pandemic, the EPA recommended the use of personal filtration systems to reduce viral air particles and individuals that obtained a personal filtration system during COVID-19 may have also used it during the smoke events of 2023 [45]. Indeed, Hoshiko et al. (2023) reported that approximately one in three people used an air purifier during times of WFS following the pandemic, in line with our findings [44].

Even though 93% of our sample took one or more actions recommended by the CDC and state DOH, 87% reported at least one WFS related health symptom. A medically vulnerable population in California similarly detailed health impacts from WFS despite taking preventative actions [44]. This suggests that it may not be enough for individuals to simply stay indoors or use masks, as draftiness of a house and mask leakage can increase exposure to the harmful components of WFS [30]. Research into mask effectiveness shows that filtration efficiency in protecting users from PM_2.5_ and WFS depends on the materials used and fit [46,47,48]. Compared to surgical masks, personal respirators offer more protection against wildfire-specific PM_2.5_ and PM_10_, but are less commonly used [47]. One study noted that the effectiveness of a mask to reduce exposure to PM_2.5_ may be limited if the mask is only worn while outside or for a short period of time [49].

We find that 60% of our sample would consider evacuating due to WFS, with 85% acknowledging it as a natural hazard. Compared to the findings of Fowler et al. (2019), a similar proportion of Idaho survey respondents view WFS as a natural hazard, but fewer would consider evacuating [28]. This suggests that NYS residents, who are not as habitually exposed to WSF, may be more likely to evacuate their homes during smoke events, but more research is needed to fully understand motivations for evacuating.

While most of our survey population viewed WFS as a hazard, experienced adverse health symptoms, and took preventative action during Summer 2023, 19% were unsure about whether they would consider evacuating in the future. Fowler et al. similarly reported significant uncertainty when considering future action surrounding WFS, with 54% viewing it as less important than other natural disasters [28]. Some individuals may perceive WFS as less of a threat compared to the flames of wildfires. A 2008 Texas-based sample noted that the majority of respondents thought that WFS was less dangerous than air pollution because it was a natural occurrence, despite the opposite being true [50]. More research is needed to understand the differences in threat perception of wildfires and WFS.

### 4.4. Implications for Public Health Practice and Policy

This work has various implications for additional research and public health practice. First, further research should focus on understanding the relative effectiveness of mitigation actions such as the use of different types of face masks and staying inside to lessen exposure to WFS. Even though most participants report taking protective measures for their health, including wearing masks, a similar number of participants reported experiencing at least one adverse symptom. Second, more research into both the mental health impacts of WFS and the perception of WFS-related illnesses is recommended. These are less studied, yet equally important areas of research, needed to effectively tailor interventions to certain populations, who may have less experience with or knowledge of the health effects of WFS. Third, interventions should focus on utilizing newer technology such as smartphone applications as a complement to existing communication methods like television or radio. Our participants reported frequent usage of smartphone applications to find and receive information about WFS. Additionally, WFS communications should be timely, engaging, easy to understand, and easy to access for all populations from younger adults to older individuals and individuals in lower socioeconomic situations. It may be helpful to include information on accessible, actionable, and effective preventative measures alongside daily WFS and air quality notifications.

### 4.5. Strengths and Limitations

While we were able to investigate a knowledge gap of perceptions and responses to WFS among NYS residents, a population historically not affected by WFS, the resulting convenience sample is not representative of the greater NYS population. However, we do provide valuable insights for a primarily well-educated, healthy, and white population of various ages and household incomes. Second, this study utilized an existing survey to maximize validity and potentially mitigate measurement error. We were able to adapt the survey quickly for rapid data collection within a few months after the smoke events occurred, with the goal of minimizing recall bias. Additionally, while we present several exploratory analyses, our study was not powered to identify high-risk populations with exposure to WFS. Therefore, the results of our exploratory analyses should be interpreted with caution and be replicated in future research. Finally, as our study was retrospective and cross-sectional, we could not assess temporal changes regarding WFS perceptions, responses, or impacts over time. Longitudinal data collection may provide a better understanding of how individuals are impacted during all stages of a smoke event, rather than just focusing on cross-sectional periods of low or high WFS and relying on recall to assess exposure. Future work should consider data collection in the same population over the course of a wildfire season to examine changes in perceptions, health, and behaviors with prolonged exposure to WFS.

## 5. Conclusions

Our study explored the ways in which a cohort of NYS residents were affected by WFS from the 2023 Canadian wildfires as well as their risk perception and actions taken to protect their health. While participants sought out or received WFS-related information and took preventative actions, a high prevalence of adverse symptoms and lifestyles changes were still reported during times of WFS. Our work provides insights on risk perceptions of a relatively WFS-naive cohort in the northeastern US, highlights the importance of timely smoke alerts, and emphasizes the need for effective public health interventions to mitigate the effects of WFS exposure.

## Figures and Tables

**Table 1:** Study Population Characteristics.

Question	Frequency (*n* = 609)	Percentage (%)
Age		
18–24	46	7.6
25–34	130	21.4
35–44	122	20
45–54	91	14.9
55–64	106	17.4
65 and over	91	14.9
Prefer not to say	23	3.8
Gender		
Woman	494	81.1
Man	103	16.9
Non-binary	9	1.5
Prefer not to say	3	0.5
Race/Ethnicity		
White	537	88.2
Hispanic/Latino	16	2.6
Black/African American	15	2.5
2+ race/ethnicity	14	2.3
Asian/Pacific Islander	13	2.1
Other	6	1
Native American/American Indian/Alaskan Native	2	0.3
Prefer not to say	6	1
Education Level		
Some high school, no diploma	2	0.3
High school graduate, diploma, or GED	30	4.9
Some college, no degree	51	8.4
Associates degree	68	11.2
Bachelor’s degree	206	33.8
Master’s degree	215	35.3
PhD, MD, JD, or similar	34	5.6
Prefer not to say	3	0.5
Household Income		
USD 25,000 or less	33	5.4
USD 25,000 to USD 49,999	91	14.9
USD 50,000 to USD 74,999	117	19.2
USD 75,000 to USD 99,999	103	16.9
USD 100,00 to USD 149,999	140	23
USD 150,000 to USD 249,999	62	10.2
USD 250,000 or more	13	2.1
Prefer not to say	50	8.2
General Health Status		
Excellent	176	28.9
Good	363	59.6
Fair	66	10.8
Poor	4	0.7
During the summer of 2023, did you experience one or more days that were smokey, or days where you felt the air quality was poor due to wildfire smoke?		
Yes	604	99.2
No	3	0.5
Not Sure	1	0.2
Prefer not to say	1	0.2
During the summer of 2023, think of the longest period of consecutive days where it was smokey where you were. How many days was it smokey?		
0 days	2	0.3
1 day	0	0
2 days	12	2
3 days	142	23.3
4 days	133	21.8
5 days	117	19.2
6 days	25	4.1
7 days or more	155	25.5
Not sure	23	3.8
During the summer of 2023, did you engage in any outdoor leisure activities, such as hiking, biking, fishing, gardening, running, or any other outdoor activity?		
Yes	544	89.3
No	63	10.3
Prefer not to say	2	0.3
During the summer of 2023, how often would you say you engaged in outdoor leisure activities you’ve listed above?		
Daily	226	37.1
A few times per week	246	40.4
Once per week	36	5.9
Less than once per week, but more than once per month	26	4.3
Rarely—A few times during the summer	9	1.5
Never	3	0.5
Prefer not to say	63	10.3
During the summer of 2023, how often did your job require you to work outdoors, exposed to any and all weather conditions?		
Daily	53	8.7
A few times per week	76	12.5
Once per week	23	3.8
Less than once per week, but more than once per month	40	6.6
Rarely—A few times during the summer	83	13.6
Never	329	54
Prefer not to say	5	0.8
During the summer of 2023, how often did your job require you to work outdoors, under cover (like in an open shed)?		
Daily	27	4.4
A few times per week	43	7.1
Once per week	10	1.6
Less than once per week, but more than once per month	27	4.4
Rarely—A few times during the summer	87	14.3
Never	411	67.5
Prefer not to say	4	0.7

**Table 2:** Dissemination of Information on Smoke Waves.

Question	Frequency (*n* = 609)	Percentage (%)
During the summer of 2023, did you ever receive any air quality notification messages related to wildfires and/or smoke events?		
Yes	558	91.6
No	39	6.4
Not sure	12	2
Which source sent you an air quality notification related to wildfires and/or smoke? [Select all that apply].		
Smartphone apps (AccuWeather, The Weather Channel, etc.)	472	77.5
Local agencies such as county health department	324	53.2
Friends or family	230	37.8
State agencies such as the Department of Environmental Conservation	164	26.9
Local news from television/radio stations	15	2.5
Other	13	2.1
Employer/workplace	10	1.6
Online air quality indexes (AirNow.gov, PurpleAir, etc.)	6	1
School/university	4	0.7
During the summer of 2023, did you ever seek out information related to wildfire and/or smoke events?		
Yes	557	91.5
No	52	8.5
Which source did you use to find wildfire smoke notifications? [Select all that apply].		
Smartphone app	397	65.2
Personal observation (seeing or smelling smoke outside)	358	58.8
Online news sources	357	58.6
Federal source such as AirNow.gov website	272	44.7
Local agencies such as county health departments	263	43.2
Social media	223	36.6
State agencies such as the Department of Environmental Conservation	174	28.6
Television	160	26.3
Friends or family	157	25.8
Newspapers	61	10
Messages or road signs on highways or interstates	55	9
Other	6	1
Online air quality indexes (PurpleAir, etc.)	5	0.8
In a smokey week in Summer 2023, approximately how many days did you look online (either on a computer, tablet, or smartphone) for smoke-related information, such as air quality, smoke forecasts, or health notices?		
0 days	14	2.3
1 day	15	2.5
2 days	37	6.1
3 days	63	10.4
4 days	58	9.5
5 days	62	10.2
6 days or more	340	55.8
Not sure	20	3.3

**Table 3:** Risk Perception of Smoke Events.

Question	Frequency (*n* = 609)	Percentage (%)
Do you consider wildfire smoke events a natural hazard?		
Yes	515	84.6
No	49	8.1
Not sure	43	7.1
Prefer not to say	2	0.3
Would you ever consider evacuating your home because of wildfire smoke (as opposed to threat from flames)?		
Yes, I would consider it.	367	60.3
No	118	19.4
Yes, I have done this in the past.	6	1
Not Sure	118	19.4

**Table 4:** Reported Health Symptoms Following Wildfire Smoke Events.

Question	Frequency (*n* = 609)	Percentage (%)
Did you experience wildfire smoke-related illness during the summer of 2023?		
Yes	150	24.6
No	374	61.4
Not sure	82	13.5
Prefer not to say	3	0.5
Did the respondent experience at least one health symptom?		
Yes	532	87.4
No	74	12.2
Prefer not to say	3	0.5
Did you have any of the following symptoms during or a few days after one of the smoke events in Summer 2023? [Select all that apply].		
Itchy, irritated, or watery eyes	385	63.2
A sore or irritated throat	302	49.6
Headache	296	48.6
Sneezing or a runny or blocked nose	203	33.3
Dry nose/sinus	151	24.8
Fatigue	129	21.2
Wheezing or whistling in chest	126	20.7
Anxiety	119	19.5
A dry cough at other times of the day	98	16.1
A dry cough at night	85	14
A dry cough first thing in the morning	66	10.8
An asthma attack	39	6.4
A wet cough (Congestion in the chest or phlegm production)	18	3
A cold	11	1.8
Other chest/lung concerns	10	1.6
Bronchitis	8	1.3
Other (Please specify)	6	1
Other head related/mental health concerns	4	1
Change in taste	2	0.3
Other throat concerns	1	0.2
Prefer not to say	77	12.6

**Table 5:** Associations between respondent characteristics and self-reported symptom prevalence and preventative action taken in New York State residents during periods of wildfire smoke in Summer 2023.

	Reported at Least 1 Symptom		Reported Headaches		Reported Itchy, Irritated, or Watery Eyes		Reported a Sore or Irritated Throat		Took Preventative Action		Would Take Future Preventative Action	
	Mean ± SD or *n* (%)	*p*	Mean ± SD or *n* (%)	*p*	Mean ± SD or *n* (%)	*p*	Mean ± SD or *n* (%)	*p*	Mean ± SD or *n* (%)	*p*	Mean ± SD or *n* (%)	*p*
Age	46.3 ± 15.8	0.0561	45.3 ± 15.0	0.132	47.0 ± 15.3	0.106	46.1 ± 15.0	0.705	45.8 ± 16.1	0.670	47.2 ± 16.4	0.313
Race/Ethnicity		0.241		0.125		0.96		**0.013**		1.00		1.00
White or Caucasian	474 (88.3%)		265 (55.2%)		340 (71.4%)		270 (57.0%)		490 (91.2%)		332 (93.5%)	
Nonwhite or non-Caucasian	43 (82.7%)		19 (43.2%)		31 (72.1%)		16 (37.2%)		48 (92.3%)		34 (94.4%)	
Gender		**0.034**		**<0.001**		0.054		**0.028**		0.642		0.177
Male	84 (81.6%)		27 (32.1%)		53 (63.1%)		38 (45.2%)		93 (90.3%)		64 (90.1%)	
Female	440 (89.1%)		261 (58.4%)		323 (73.4%)		256 (58.2%)		453 (91.7%)		306 (94.4%)	
Reported Health		0.207		**0.001**		0.203		0.896		0.112		0.298
Excellent	150 (85.2%)		68 (43.6%)		102 (68.0%)		84 (56.0%)		156 (88.6%)		114 (91.1%)	
Good/Fair/Poor	385 (88.9%)		228 (59.1%)		283 (73.5%)		218 (56.6%)		401 (92.6%)		265 (94.6%)	
Household Income		0.692		0.66		**0.017**		0.463		0.417		0.15
USD 25,000 and under	29 (87.9%)		19 (65.5%)		27 (93.1%)		13 (44.8%)		32 (97%)		21 (95.5%)	
USD 25,001 to USD 49,999	82 (90.1%)		48 (57.8%)		55 (67.1%)		48 (58.5%)		86 (94.5%)		58 (92.1%)	
USD 50,000 to USD 149,999	316 (87.8%)		174 (54.4%)		217 (68.7%)		176 (55.7%)		326 (90.6%)		219 (95.2%)	
USD 150,000 or more	63 (84.0%)		35 (53.8%)		48 (76.2%)		39 (61.9%)		67 (89.3%)		47 (87%)	

Associations between respondent characteristics and outcomes taken were assessed using *t*-tests, Fisher’s exact tests, and Chi-square tests, comparing those that reported outcomes to those that did not. Analyses reported here excluded missing, unknown, and unsure responses. Bolded *p*-values reflect statistical significance at a threshold of *p* < 0.05.

**Table 6:** Mitigation Actions Taken During Smoke Events.

Question	Frequency (*n* = 609)	Percentage (%)
During the summer of 2023, did you ever reduce or eliminate your outside activities due to wildfire smoke?		
Yes	565	92.8
No	42	6.9
Not Sure	1	0.2
Prefer not to say	1	0.2
During the summer of 2023, think of the longest period of consecutive days you reduced or eliminated your outdoor activities due to a smoke event. How many consecutive days did you reduce or eliminate activity?		
0 days	29	4.8
1 day	37	6.1
2 days	105	17.2
3 days	143	23.5
4 days	95	15.6
5 days	73	12
6 days or more	98	16.1
Not sure	21	3.5
Prefer not to say	8	1.3
If you decided to limit or eliminate your outdoor activity during a smoke event, what type of information motivated your decision to do so? [Click on all that apply].		
Your own observation (seeing or smelling smoke outside)	490	80.5
Air quality information from local, state, or federal sources	486	79.8
Smoke forecasts	235	38.6
Statistics on smoke-related health problems	207	34
Advice from family and friends	65	10.7
Advice from your doctor	39	6.4
Personal effects of smoke	10	1.6
Medical conditions of self and household	10	1.6
Other	8	1.3
Smoke data	4	0.7
Peer example	3	0.5
What is the minimum air quality index rating that would cause you to reduce your outdoor activity on a particular day?		
Green—Good	4	0.7
Yellow—Moderate	124	20.4
Orange—unhealthy for sensitive groups	306	50.3
Red—Unhealthy	120	19.7
Purple—Very unhealthy	24	3.9
Maroon—Hazardous	13	2.1
I am not familiar with this rating	11	1.8
Prefer not to say	7	1.2
What is the minimum air quality index rating that would cause you to eliminate your outdoor activity on a particular day?		
Green—Good	2	0.3
Yellow—Moderate	34	5.6
Orange—unhealthy for sensitive groups	208	34.2
Red—Unhealthy	250	41.1
Purple—Very unhealthy	67	11
Maroon—Hazardous	36	5.9
I am not familiar with this rating	11	1.8
Prefer not to say	1	0.2
Did you take any of the following actions during or following a smoke event in Summer 2023? [Select all that apply].		
Avoid outdoor leisure activities	485	75.2
Wear a mask to protect your lungs	327	53.7
Use a personal air filtration system in your home or office	208	34.2
Take medication to alleviate smoke-related symptoms	128	21
Go to buildings that have air filtration systems like the mall or library	63	10.3
Miss work or other commitments due to potential health concerns	60	9.9
Visit or consult a healthcare provider for other health concerns	28	4.6
Visit or consult a healthcare provider for asthma or smoke-related lung issues	23	3.8
Other	11	1.8
Whole house AC	2	0.3
Prefer not to say	52	8.5
Will you take preventative action to reduce smoke-related health impacts in the future?		
Yes	379	62.2
No	25	4.1
Not sure	197	32.4
Prefer not to say	8	1.3

## Data Availability

The raw data supporting the conclusions of this article will be made available by the authors on request.

## Supplementary Materials

The following supporting information can be downloaded at: https://www.mdpi.com/article/10.3390/ijerph22020277/s1, Table S1: Associations between respondent characteristics and self-reported symptom prevalence in New York State residents during periods of wildfire smoke in summer 2023; Table S2: Associations between respondent characteristics and self-reported itchy/irritated/watery eyes in New York State residents during periods of wildfire smoke in summer 2023; Table S3: Associations between respondent characteristics and self-reported sore/irritated throat in New York State residents during periods of wildfire smoke in summer 2023; Table S4: Associations between respondent characteristics and self-reported headaches in New York State residents during periods of wildfire smoke in summer 2023; Table S5: Associations between respondent characteristics and mitigation actions taken by New York State residents during periods of wildfire smoke in summer 2023; Table S6: Associations between respondent characteristics and intention to take future preventative action in New York State residents following periods of wildfire smoke in summer 2023; File S1: Survey Questionnaire.
