# Supplementary material for: Perceptions of and Responses to Wildfire Smoke Among New York State Residents: A Cross-Sectional Study
Source: Int J Environ Res Public Health. 2025 Feb 14;22(2):277. doi: 10.3390/ijerph22020277 (PMC11855130; doi:10.3390/ijerph22020277)
Supplement: Supplementary file 1 [file ijerph-22-00277-s001.zip › ijerph-3385729-supplementary.pdf]

Supplementary Information for

Perceptions of and Responses to Wildfire Smoke Among New York State Residents: A Cross-Sectional Study

Erandy Barrera, Alistair Hayden, Genevive Meredith and Corinna A. Noel *

Supplementary Table S1. Associations between respondent characteristics and self-reported symptom prevalence in New York State residents during periods of wildfire smoke in summer 2023

	Total	Did not report any symptoms	Reported at least 1 symptom	*p*-Value
	N	Mean ± SD or N (%)	Mean ± SD or N (%)	
**Age** [a]	586	42.45 ± 15.73	46.32 ± 15.79	0.0561
**Race/Ethnicity** [b]				
White or Caucasian	537	63 (11.7%)	474 (88.3%)	0.241
Nonwhite or non-Caucasian	52	9 (17.3%)	43 (82.7%)	
**Gender** [b]				
Male	103	19 (18.4%)	84 (81.6%)	**0.034**
Female	494	54 (10.9%)	440 (89.1%)	
**Reported Health Status** [b]				
Excellent	176	26 (14.8%)	150 (85.2%)	0.207
Good/Fair/Poor	433	48 (11.1%)	385 (88.9%)	
**Household Income** [c]				
$25,000 and under	33	4 (12.1%)	29 (87.9%)	0.692
$25,001 to $49,999	91	9 (9.9%)	82 (90.1%)	
$50,000 to $149,999	360	44 (12.2%)	316 (87.8%)	
$150,000 or more	75	12 (16.0%)	63 (84.0%)	

Associations between respondent characteristics and symptom prevalence were assessed using the following analyses: [a] T-test, [b] Chi-square test, and [c] Fisher's exact test. Analyses reported here excluded missing, unknown, and unsure responses. Bolded *p*-values reflect statistical significance at a threshold of $p<0.05$.

Supplementary Table S2. Associations between respondent characteristics and self-reported itchy/irritated/watery eyes in New York State residents during periods of wildfire smoke in summer 2023

	Total	Did not report itchy, irritated, or watery eyes	Reported itchy, irritated, or watery eyes	*p*-Value
	N	Mean ± SD or N (%)	Mean ± SD or N (%)	
**Age** [a]	517	44.54 ± 16.84	47.03 ± 15.32	0.106
**Race/Ethnicity** [b]				
White or Caucasian	474	134 (28.3%)	340 (71.4%)	0.960
Nonwhite or non-Caucasian	43	12 (27.9%)	31 (72.1%)	
**Gender** [b]				
Male	84	31 (36.9%)	53 (63.1%)	0.054
Female	440	117 (26.6%)	323 (73.4%)	
**Reported Health Status** [b]				
Excellent	150	48 (32.0%)	102 (68.0%)	0.203
Good/Fair/Poor	385	102 (26.5%)	283 (73.5%)	
**Household Income** [c]				
$25,000 and under	29	2 (6.9%)	27 (93.1%)	
$25,001 to $49,999	82	27 (32.9%)	55 (67.1%)	**0.017**
$50,000 to $149,999	316	99 (31.3%)	217 (68.7%)	
$150,000 or more	63	15 (23.8%)	48 (76.2%)	

Associations between respondent characteristics and symptom prevalence were assessed using the following analyses: [a] T-test, [b] Chi-square test, and [c] Fisher's exact test. Analyses reported here excluded missing, unknown, and unsure responses. Bolded *p*-values reflect statistical significance at a threshold of $p < 0.05$.

Supplementary Table S3. Associations between respondent characteristics and self-reported sore/irritated throat in New York State residents during periods of wildfire smoke in summer 2023

	Total	Did not report a sore or irritated throat	Reported a sore or irritated throat	p-Value
	**N**	**Mean ± SD or N (%)**	**Mean ± SD or N (%)**	
**Age** [a]	517	46.62 ± 16.79	46.09 ± 15.01	0.705
**Race/Ethnicity** [b]				
White or Caucasian	474	204 (43.0%)	270 (57.0%)	0.013
Nonwhite or non-Caucasian	43	27 (62.8%)	16 (37.2%)	
**Gender** [b]				
Male	84	46 (54.8%)	38 (45.2%)	**0.028**
Female	440	184 (41.8%)	256 (58.2%)	
**Reported Health Status** [b]				
Excellent	150	66 (44.0%)	84 (56.0%)	0.896
Good/Fair/Poor	385	167 (43.4%)	218 (56.6%)	
**Household Income** [b]				
$25,000 and under	29	16 (55.2%)	13 (44.8%)	
$25,001 to $49,999	82	34 (41.5%)	48 (58.5%)	0.463
$50,000 to $149,999	316	140 (44.3%)	176 (55.7%)	
$150,000 or more	63	24 (38.1%)	39 (61.9%)	

Associations between respondent characteristics and symptom prevalence were assessed using the following analyses: [a] T-test and [b] Chi-square test. Analyses reported here excluded missing, unknown, and unsure responses, leading to inconsistent sample sizes. Bolded p-values reflect statistical significance at a threshold of $p<0.05$.

Supplementary Table S4. Associations between respondent characteristics and self-reported headaches in New York State residents during periods of wildfire smoke in summer 2023

	Total	Did not report headaches	Reported headaches	
	**N**	**Mean ± SD or N (%)**	**Mean ± SD or N (%)**	***p*-Value**
**Age** [a]	523	47.37 ± 16.59	45.29 ± 14.95	0.132
**Race/Ethnicity** [b]				
White or Caucasian	480	215 (44.8%)	265 (55.2%)	0.125
Nonwhite or non-Caucasian	44	25 (56.8%)	19 (43.2%)	
**Gender** [b]				
Male	84	57 (67.9%)	27 (32.1%)	**<0.001**
Female	447	186 (41.6%)	261 (58.4%)	
**Reported Health Status** [b]				
Excellent	156	88 (56.4%)	68 (43.6%)	**0.001**
Good/Fair/Poor	386	158 (40.9%)	228 (59.1%)	
**Household Income** [b]				
$25,000 and under	29	10 (34.5%)	19 (65.5%)	
$25,001 to $49,999	83	35 (42.2%)	48 (57.8%)	0.660
$50,000 to $149,999	320	146 (45.6%)	174 (54.4%)	
$150,000 or more	65	30 (46.2%)	35 (53.8%)	

Associations between respondent characteristics and symptom prevalence were assessed using the following analyses: [a] T-test and [b] Chi-square test. Analyses reported here excluded missing, unknown, and unsure responses, leading to inconsistent sample sizes. Bolded p-values reflect statistical significance at a threshold of p<0.05.

Supplementary Table S5. Associations between respondent characteristics and mitigation actions taken by New York State residents during periods of wildfire smoke in summer 2023

	Total	Did not take preventative action	Took preventative action	
	N	Mean ± SD or N (%)	Mean ± SD or N (%)	*p*-Value
**Age** [a]	586	46.81 ± 12.88	45.78 ± 16.06	0.670
**Race/Ethnicity** [b]				
White or Caucasian	537	47 (8.8%)	490 (91.2%)	1.000
Nonwhite or non-Caucasian	52	4 (7.7%)	48 (92.3%)	
**Gender** [c]				
Male	103	10 (9.7%)	93 (90.3%)	0.642
Female	494	41 (8.3%)	453 (91.7%)	
**Reported Health Status** [c]				
Excellent	176	20 (11.4%)	156 (88.6%)	0.112
Good/Fair/Poor	433	32 (7.4%)	401 (92.6%)	
**Household Income** [b]				
$25,000 and under	33	1 (3%)	32 (97%)	
$25,001 to $49,999	91	5 (5.5%)	86 (94.5%)	0.417
$50,000 to $149,999	360	34 (9.4%)	326 (90.6%)	
$150,000 or more	75	8 (10.7%)	67 (89.3%)	

Associations between respondent characteristics and preventative action taken were assessed using the following analyses: [a] T-test, [b] Fisher's exact test, and [c] Chi-square test. Analyses reported here excluded missing, unknown, and unsure responses, leading to inconsistent sample sizes.

Supplementary Table S6. Associations between respondent characteristics and intention to take future preventative action in New York State residents following periods of wildfire smoke in summer 2023

	Total	Would not take future preventative action	Would take future preventative action	*p*-Value
	N	Mean ± SD or N (%)	Mean ± SD or N (%)	
**Age** [a]	391	43.52 ± 12.08	47.19 ± 16.37	0.313
**Race/Ethnicity** [b]				
White or Caucasian	355	23 (6.5%)	332 (93.5%)	1.000
Nonwhite or non-Caucasian	36	2 (5.6%)	34 (94.4%)	
**Gender** [c]				
Male	71	7 (9.9%)	64 (90.1%)	0.177
Female	324	18 (5.6%)	306 (94.4%)	
**Reported Health Status** [c]				
Excellent	124	10 (8.1%)	114 (91.1%)	0.298
Good/Fair/Poor	280	15 (5.4%)	265 (94.6%)	
**Household Income** [b]				
$25,000 and under	22	1 (4.5%)	21 (95.5%)	0.150
$25,001 to $49,999	63	5 (7.9%)	58 (92.1%)	
$50,000 to $149,999	230	11 (4.8%)	219 (95.2%)	
$150,000 or more	54	7 (13%)	47 (87%)	

Associations between respondent characteristics and intention to take future preventative action were assessed using the following analyses: [a] T-test, [b] Fisher's exact test, and [c] Chi-square test. Analyses reported here excluded missing, unknown, and unsure responses, leading to inconsistent sample sizes.

Supplementary File S1. Survey Questionnaire

Title: Wildfire Smoke Project

1. **Informed Consent Form**

   Share your experience with wildfire smoke during summer 2023.

   The goal of this research is to understand how individuals in the Northeastern United States perceive and respond to wildfire smoke, particularly in the context of the summer 2023 wildfire season. We are asking you to take part in our online survey, which will take approximately 10 minutes to complete. In a series of multiple choice questions, we will ask you how you typically access information on air quality, if you have experienced periods of poor air quality due to smoke, how smoke affects you, and how your behaviors change during times of heavy smoke. Results from this research will contribute to greater shared knowledge with the goal of advancing efforts related to wildfire smoke mitigation and response in the United States.

   When you complete the survey, you will have the opportunity to enter a raffle for a chance to win one of five $50 Amazon gift cards. Winners will be randomly selected by November 15, 2023 and prizes will be sent via mail. Any identifiable information provided by those entering the raffle will not be connected to survey responses.

   We do not anticipate any risks or benefits from participating in this survey. Taking part in this survey is completely voluntary, and you may refuse to participate at this time or at any point in the future. You may also skip any questions/procedures that may make you feel uncomfortable, with no penalty to you. If you have questions now or later, you may contact Corinna Noel, Assistant Professor of Practice at Cornell University at can64@cornell.edu. If you have any questions or concerns regarding your rights as a participant, you may contact the Institutional Review Board (IRB) for Human Participants at 607-255-5138 or anonymously at 1- 866-293-3077.

   De-identified data from this survey may be shared with key stakeholders and the larger public and environmental health community to advance efforts related to wildfire smoke mitigation and response in the United States. No one will be able to

identify you from the information we share.

By clicking "next" below, you are confirming that you understand what we are asking of you and agree to participate. You also certify that you are 18+ years old and spent the majority of your time in the Northeastern United States (including Connecticut, Delaware, District of Columbia, Maine, Maryland, Massachusetts, New Hampshire, New Jersey, New York, Pennsylvania, Rhode Island, Vermont, Virginia, or West Virginia) during the summer of 2023.

2. **Natural Hazard Questions (2 questions)**

2.1. Do you consider wildfire smoke events a natural hazard?

a. Yes
b. No
c. Not sure

2.2. Would you ever consider evacuating your home because of wildfire smoke (as opposed to threat from flames)?

a. Yes, I have done this in the past.
b. Yes, I would consider it.
c. No
d. Not sure

3. **Air Quality Notification (9 Questions)**

3.1. During the summer of 2023, where did you spend a majority of your time?

a. Select a state (Drop down)

3.2. During the summer of 2023, did you experience one or more days that were smoky, or days where you felt the air quality was poor due to wildfire smoke?

a. Yes
b. No
c. Not sure

3.3. During the summer of 2023, think of the longest period of consecutive days where it was smoky where you were. How many consecutive days was it smokey?

a. 0 days
b. 1 day
c. 2 days
d. 3 days

e. 4 days
f. 5 days
g. 6 days
h. 7 days or more
i. Not sure

3.4. During the summer of 2023, did you ever receive any air quality notification messages related to wildfires and/or smoke events?

a. Yes
b. No
c. Not sure

3.5. Which source sent you an air quality notification related to wildfires and/or smoke? [Click on all that apply]

a. Friends or family
b. Smartphone app (Accuweather, The Weather Channel, etc.)
c. Local agencies such as county health department
d. State agencies such as the Department of Environmental Conservation
e. Other (please specify)

3.6. During the summer of 2023, did you ever seek out information related to wildfire and/or smoke events?

a. Yes
b. No

3.7. Which source did you use to find wildfire smoke notifications? [Click on all that apply]

a. Social media (Facebook, Instagram, Twitter, etc.)
b. Television
c. Online news sources
d. Newspapers
e. Friends or family
f. Messages or road signs on highways or interstates
g. Personal observation (seeing or smelling smoke outside)
h. Smartphone app (Accuweather, The Weather Channel, etc.)
i. Local agencies such as county health department
j. State agencies such as the Department of Environmental Conservation
k. Federal source such as AIRnow.gov website

l. Other (please specify)

3.8. In a smoky week in summer 2023, approximately how many days did you look online (either on a computer, tablet, or smartphone) for smoke-related information, such as air quality, smoke forecasts, or health notices?

a. 0 days
b. 1 day
c. 2 days
d. 3 days
e. 4 days
f. 5 days
g. 6 days or more
h. Not sure

3.9. If you decided to limit or eliminate your outdoor activity during a smoke event, what type of information motivated your decision to do so? [Click on all that apply]

a. Statistics on smoke-related health problems
b. Air quality information from local, state, or federal sources
c. Smoke forecasts
d. Your own observation (seeing or smelling smoke outside)
e. Advice from your doctor
f. Advice from family and friends
g. Other (please specify)

4. Activity Data (8 questions)

4.1. During the summer of 2023, did you engage in any outdoor leisure activities, such as hiking, biking, fishing, gardening, running, or any other outdoor activity?

a. Yes – Please list the activities
b. No

4.2. During the summer of 2023, how often would you say you engaged in outdoor leisure activities you've listed above?

a. Daily
b. A few times per week
c. Once per week
d. Less than once per week, but more than once per month

    e. Rarely – A few times during the summer
    f. Never

4.3. During the summer of 2023, how often did your job require you to work outdoors, exposed to any and all weather conditions?
    a. Daily
    b. A few times per week
    c. Once per week
    d. Less than once per week, but more than once per month
    e. Rarely – A few times during the summer
    f. Never

4.4. During the summer of 2023, how often did your job require you to work outdoors, under cover (like in an open shed)?
    a. Daily
    b. A few times per week
    c. Once per week
    d. Less than once per week, but more than once per month
    e. Rarely – A few times during the summer
    f. Never

4.5. During the summer of 2023, did you ever reduce or eliminate your outside activities due to wildfire smoke?
    a. Yes
    b. No
    c. Not sure

4.6. During the summer of 2023, think of the longest period of consecutive days you reduced or eliminated your outdoor activities due to a smoke event. How many consecutive days did you reduce or eliminate activity?
    a. 0 days
    b. 1 day
    c. 2 days
    d. 3 days
    e. 4 days
    f. 5 days
    g. 6 days or more
    h. Not sure

4.7. What is the minimum air quality index rating that would cause you to reduce your outdoor activity on a particular day?
   a. Green – good
   b. Yellow – moderate
   c. Orange – Unhealthy for sensitive groups
   d. Red – Unhealthy
   e. Purple – Very unhealthy
   f. Maroon – Hazardous
   g. I am not familiar with this rating

4.8. What is the minimum air quality index rating that would cause you to eliminate your outdoor activity on a particular day?
   a. Green – good
   b. Yellow – moderate
   c. Orange – Unhealthy for sensitive groups
   d. Red – Unhealthy
   e. Purple – Very unhealthy
   f. Maroon – Hazardous
   g. I am not familiar with this rating

5. **Health Questions (5 questions)**

5.1. In general, would you say that your health is:
   a. Excellent
   b. Good
   c. Fair
   d. Poor

5.2. Did you experience wildfire smoke-related illness during the summer of 2023?
   a. Yes
   b. No
   c. Not sure

5.3. Did you have any of the following symptoms during or a few days after one of the smoke events in summer 2023? [Click on all that apply]
   a. Wheezing or whistling in chest
   b. Itchy, irritated, or watery eyes
   c. Sneezing or a runny or blocked nose
   d. Dry nose / sinus

e. A sore or irritated throat
f. A cold
g. A dry cough at night
h. A dry cough first thing in the morning
i. A dry cough at other times of the day
j. A wet cough (congestion in the chest or phlegm production)
k. Bronchitis
l. An asthma attack
m. Headache
n. Fatigue
o. Anxiety
p. Other (please specify)

5.4. Did you take any of the following actions during or following a smoke event in summer 2023? [Click on all that apply]

a. Take medication to alleviate smoke-related symptoms
b. Wear a mask to protect your lungs
c. Visit or consult a healthcare provider for asthma or smoke-related lung issues
d. Visit or consult a healthcare provider for other health concerns
e. Use a personal air filtration system in your home or office
f. Avoid outdoor leisure activities
g. Go to buildings that have air filtration systems like the mall or public library
h. Miss work or other commitments due to potential health concerns
i. Other (please specify)

5.5. Will you take preventative action to reduce smoke-related health impacts in the future?

a. Yes – Please list all the actions you might take
b. No
c. Not sure

6. Demographic Data (6 questions)

6.1. What is your age?

a. (Write in box)

6.2. Which gender do you identify with?

a. Woman
b. Man

c. Other

6.3. What racial or ethnic group best describes you?

a. White/Caucasian
b. Hispanic or Latino
c. Black or African American
d. Native American / American Indian or Alaskan Native
e. Asian / Pacific Islander
f. Other

6.4. Where did you live during summer 2023?

a. City/Town
b. State [Has verification]
c. Zip code [Has verification]
d. County

6.5. What is the highest degree or level of school you completed? If currently enrolled, highest degree received.

a. 8th grade or less
b. Some high school, no diploma
c. High school graduate, diploma, or GED
d. Some college, no degree
e. Associates degree
f. Bachelor's degree
g. Master's degree
h. Ph.D., M.D., J.D., or similar

6.6. What is your total household income, including income from all members of your family, in 2023 before taxes? This figure should include salaries, wages, pensions, dividends, interest, and all other income.

a. $25,000 or less
b. $25,000 to $49,999
c. $50,000 to $74,999
d. $75,000 to $99,999
e. $100,000 to $149,999
f. $150,000 to $249,999
g. $250,000 or more

7. Survey Raffle Entry (2 questions)

7.1. Would you like to enter a raffle for a chance to win a $50 Amazon gift card? Please note that you must fall within our study region (Northeastern US) to be eligible to receive the gift card via mail.

   a. Yes
   b. No

7.2. Please provide an email for raffle entry. We will randomly pick winners on November 15, 2023.

   a. (Write in box)

8. End message

Thank you for taking the time to complete the survey!

We appreciate your responses.

They will contribute to the improvement of emergency preparedness strategies and policies to address the impact of the Summer 2023 wildfire smoke on residents of Northeastern United States.
